# BATF sustains homeostasis and functionality of bone marrow Treg cells to preserve homeostatic regulation of hematopoiesis and development of B cells

**DOI:** 10.3389/fimmu.2023.1026368

**Published:** 2023-02-22

**Authors:** Chiranjeevi Tikka, Lindsay Beasley, Chengxian Xu, Jing Yang, Scott Cooper, Joseph Lechner, Sarah Gutch, Mark H. Kaplan, Maegan Capitano, Kai Yang

**Affiliations:** ^1^ Department of Pediatrics and the Herman B Wells Center for Pediatric Research, Indiana University School of Medicine, Indianapolis, IN, United States; ^2^ Department of Microbiology and Immunology, Indiana University School of Medicine, Indianapolis, IN, United States

**Keywords:** Treg cells, BATF, IL-7, bone marrow, hematopoiesis, B cell development

## Abstract

Bone marrow Treg cells (BM Tregs) orchestrate stem cell niches crucial for hematopoiesis. Yet little is known about the molecular mechanisms governing BM Treg homeostasis and function. Here we report that the transcription factor BATF maintains homeostasis and functionality of BM Tregs to facilitate homeostatic regulation of hematopoiesis and B cell development. Treg-specific ablation of BATF profoundly compromised proportions of BM Tregs associated with reduced expression of Treg effector molecules, including CD44, ICOS, KLRG1, and TIGIT. Moreover, BATF deficiency in Tregs led to increased numbers of hematopoietic stem cells (HSCs), multipotent progenitors (MPPs), and granulocyte-macrophage progenitors (GMPs), while reducing the functionality of myeloid progenitors and the generation of common lymphoid progenitors. Furthermore, Tregs lacking BATF failed to support the development of B cells in the BM. Mechanistically, BATF mediated IL-7 signaling to promote expression of effector molecules on BM Tregs and their homeostasis. Our studies reveal a previously unappreciated role for BATF in sustaining BM Treg homeostasis and function to ensure hematopoiesis.

## Introduction

Aside from the importance of FOXP3^+^ regulatory T cells (Tregs) in maintaining immune tolerance (1, 2), emerging evidence highlights that Tregs are crucial to maintain self-renewal and multi-lineage differentiation capacity of stem cells in different tissues (3, 4). In the bone marrow (BM) following allogenic BM transplantation, Tregs co-localize with infused allogeneic hematopoietic stem cells (HSCs) creating unique niches to maintain their quiescence and development (3, 5). In comparison to the spleen, the BM contains a higher frequency of Tregs (6, 7), which have greater expression of Treg effector molecules (8). By secreting high levels of IL-10, BM Tregs maintain homeostasis and function of mesenchymal stromal cells (MSCs) and HSCs (7). In addition, BM Tregs are indispensable for B cell lymphopoiesis through sustaining a subpopulation of ICAM1^+^ stromal cells that produce IL-7 (9). Despite these associations, the signaling link underlying homeostasis and functionality of BM Tregs in regulating hematopoiesis has yet to be established.

Tregs coordinate microenvironmental immune signals to orchestrate transcriptional networks in support of Treg cell homeostasis and functionality in various lymphoid and non-lymphoid tissues (10). The basic leucine zipper ATF-like transcription factor (BATF), a member of the AP-1 transcription factor family, plays a crucial role in driving the differentiation and function of a variety of T cell subsets (11–14). In Tregs, the initial study revealed that Tregs need BATF to induce expression of gut-homing receptors and hence promotes suppressive function in preventing colitis (15). More recently, several lines of evidence highlight that BATF promotes maturation and homeostasis of tissue-resident Tregs (16, 17) by remodeling DNA-methylation landscape and chromatin accessibility to facilitate the generation of non-lymphoid Treg precursors (18, 19). BATF cooperates with FOXP3 to support the differentiation and accumulation of tissue Tregs (20). Upon antigen stimulation, BATF coordinates functional specification and fitness of triglyceride metabolism of Tregs to restrain allergic inflammation (21). In adipose tissues, BATF integrates microenvironmental cytokine IL-33 to trigger unique transcriptional programs underlying homeostasis and functionality of adipose tissue Tregs (16). However, whether BATF regulates homeostasis and functionality of BM Tregs in hematopoiesis remains to be defined.

Here we demonstrate a previously unappreciated role for BATF in maintaining homeostasis and functionality of BM Tregs in support of hematopoiesis and B cell development. Treg-specific deletion of BATF markedly compromised proportions of BM Tregs without substantially affecting those in the spleen. Despite the reduction of BATF-deficient BM Tregs, the BM did not display overt inflammatory responses. BATF deficiency abrogated the generation of BM effector Tregs and their homeostatic proliferation. Moreover, analysis in BM of *Foxp3* ^Cre^ *Batf* ^fl/fl^ mice demonstrated increased numbers of hematopoietic stem cells and multiple myeloid lineage progenitors, but impaired capacity of their expansion and colonization *ex vivo*. Transplantation of *Foxp3* ^Cre^ *Batf* ^fl/fl^ BM into lethally irradiated recipient mice demonstrated an increase in short-term recovery but an overall defect in myeloid cell recovery. Under homeostatic conditions BATF-deficient Tregs failed to maintain the proportions of common lymphoid progenitors and support B cell lymphopoiesis. Mechanistically, loss of BATF impaired maintenance of BM Tregs and their upregulation of Treg effector molecules, including CD44, ICOS, KLRG1, and TIGIT in response to IL-7 stimulation. Collectively, our studies provide mechanistic insights underlying Treg-dependent regulation of hematopoiesis in BM.

## Results

### IL-7 facilitates Tregs to acquire effector markers in the BM

**Figure 1:**
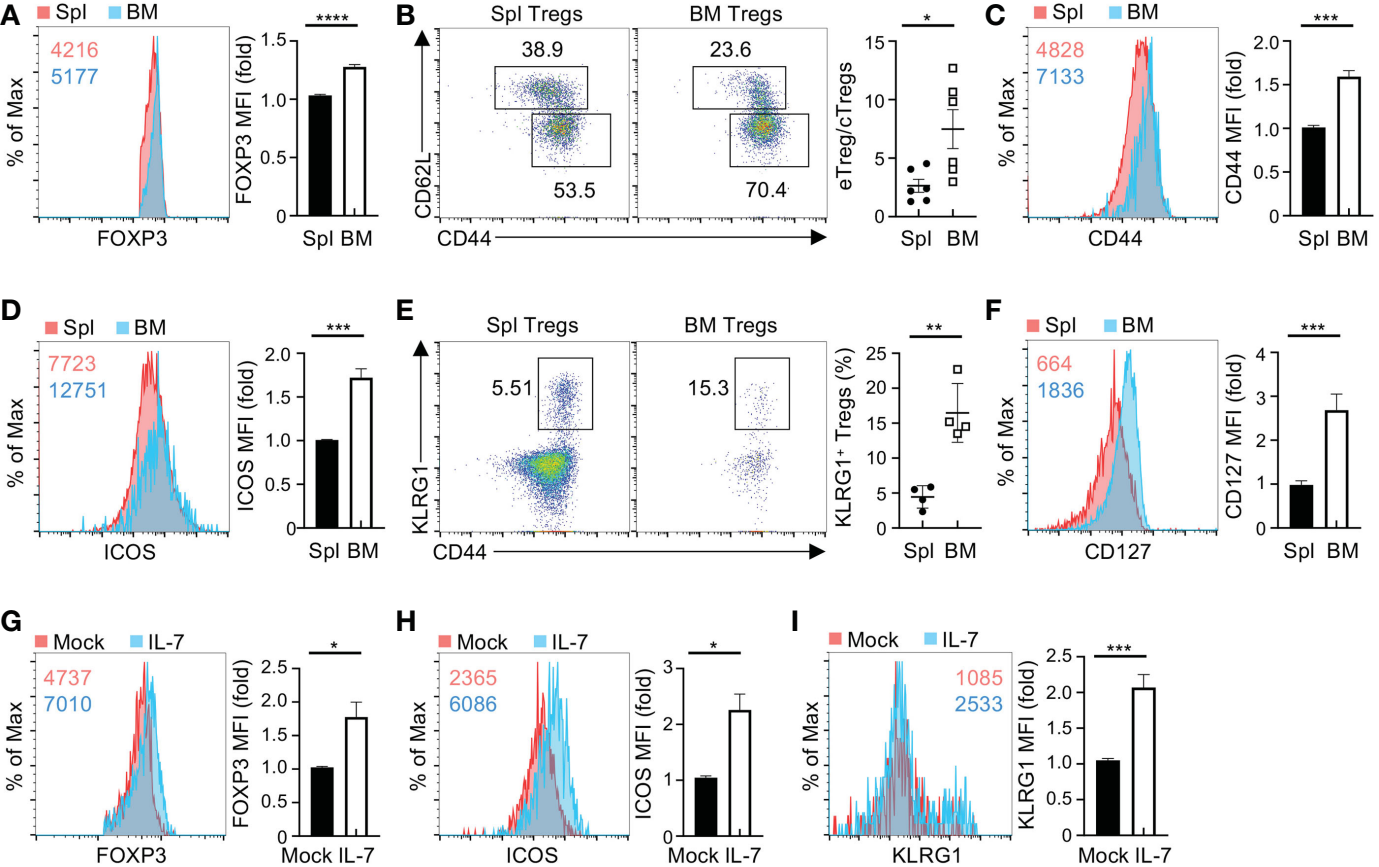
BM Tregs express high levels of CD127 and eTreg-associated markers. **(A)** Flow cytometry analysis of FOXP3 expression in Tregs from the spleen (Spl) and BM of *Foxp3* ^Cre^ mice. Numbers in graph plot showing mean fluorescence intensity (MFI) of FOXP3. Right, fold changes of FOXP3 MFI in spleen and BM Tregs. **(B)** Expression of CD62L and CD44 in spleen and BM Tregs from *Foxp3* ^Cre^ mice. Right, ratios of CD44^hi^ Tregs to CD44^lo^ Tregs (*n* = 6 per group). **(C, D)** Expression of CD44 **(C)** and ICOS **(D)** in spleen and BM Tregs from *Foxp3* ^Cre^ mice. Right, fold changes of CD44 **(C)** and ICOS **(D)** MFI in spleen and BM Tregs. **(E)** Flow cytometry analysis of KLRG1^+^CD44^+^ Tregs in the spleen and BM from *Foxp3* ^Cre^ mice. Right, frequencies of KLRG1^+^CD44^+^ Tregs (*n* = 4 per group). **(F)** Expression of CD127 on spleen and BM Tregs from *Foxp3* ^Cre^ mice. Right, fold changes of CD127 MFI in spleen and BM Tregs. **(G-I)** Expression of FOXP3 **(G)**, ICOS **(H)**, and KLRG1 **(I)** in *Foxp3* ^Cre^ BM Tregs with mock or IL-7 stimulation. Right, fold changes of FOXP3 **(G)**, ICOS **(H)**, and KLRG1 **(I)** MFI in BM Tregs with mock or IL-7 stimulation. Data are representative of at least three independent experiments **(A-I)**. Data mean ± s.e.m. *P* values are determined by two-tailed Student’s *t*-test **(A-I)**. *P < 0.05, **P < 0.01, ***P < 0.001, and ****P < 0.0001.

BM Tregs play a crucial role in maintaining quiescence of HSCs (3, 7) and B cell lymphopoiesis (9), whereas the molecular mechanism governing BM Treg homeostasis and function remains elusive. To explore signaling pathways involved in the process, we examined proportions of Tregs in the BM and spleen from *Foxp3* ^Cre^ mice (designated as WT mice). Of note, BM showed higher frequencies of Tregs than spleen (**Figure S1A**), concomitant with an increased expression of FOXP3 (**Figure 1A**). Based on expression of CD62L and CD44, Tregs can be defined as central Tregs (cTregs, CD62L^hi^CD44^lo^) and effector Tregs (eTregs, CD62^lo^CD44^hi^) (22). Compared to splenic Tregs, BM Tregs had higher frequencies of eTregs and reciprocally lower proportions of cTregs (**Figure 1B**), leading to the heightened ratio of eTregs to cTregs in the BM (**Figure 1B**). Consistent with these observations, BM Tregs showed increased expression of eTreg-associated molecules including CD44 (**Figure 1C**), ICOS (**Figure 1D**), and KLRG1 (**Figure 1E**). These results indicate that Tregs acquire eTreg-associated features in the BM.

Microenvironmental cytokines are crucial to maintain homeostasis and functionality of tissue-resident Tregs (23). Given the notion that IL-2 and IL-7 differentially regulate the maintenance of Tregs in different tissues (22, 24), we examined the expression of their receptors CD25 and CD127 on BM and spleen Tregs, respectively. Compared to splenic Tregs, BM Tregs showed reduced expression of CD25 (**Figure S1B**), while significantly increasing the expression of CD127 (**Figure 1F**). These observations suggested that IL-7 potentially regulates BM Treg homeostasis. We thus assessed whether IL-7 influences BM Treg maintenance and expression of eTreg-associated markers. To this end, we enriched BM Tregs and then stimulated them with IL-7 for 2 days. IL-7 stimulation elevated the expression of FOXP3 (**Figure 1G**) and eTreg-associated markers including ICOS (**Figure 1H**), and KLRG1 (**Figure 1I**) in BM Tregs. In contrast, IL-7 did not substantially induce the expression of CD44 (**Figure S1C**) or CXCR4 (**Figure S1D**), which mediates traffic of Tregs into the BM (25). Collectively, these results imply that BM Tregs integrate microenvironmental IL-7 to promote the expression of eTreg-associated markers.

### BATF deficiency impairs BM Treg homeostasis and expression of eTreg-associated markers

**Figure 2:**
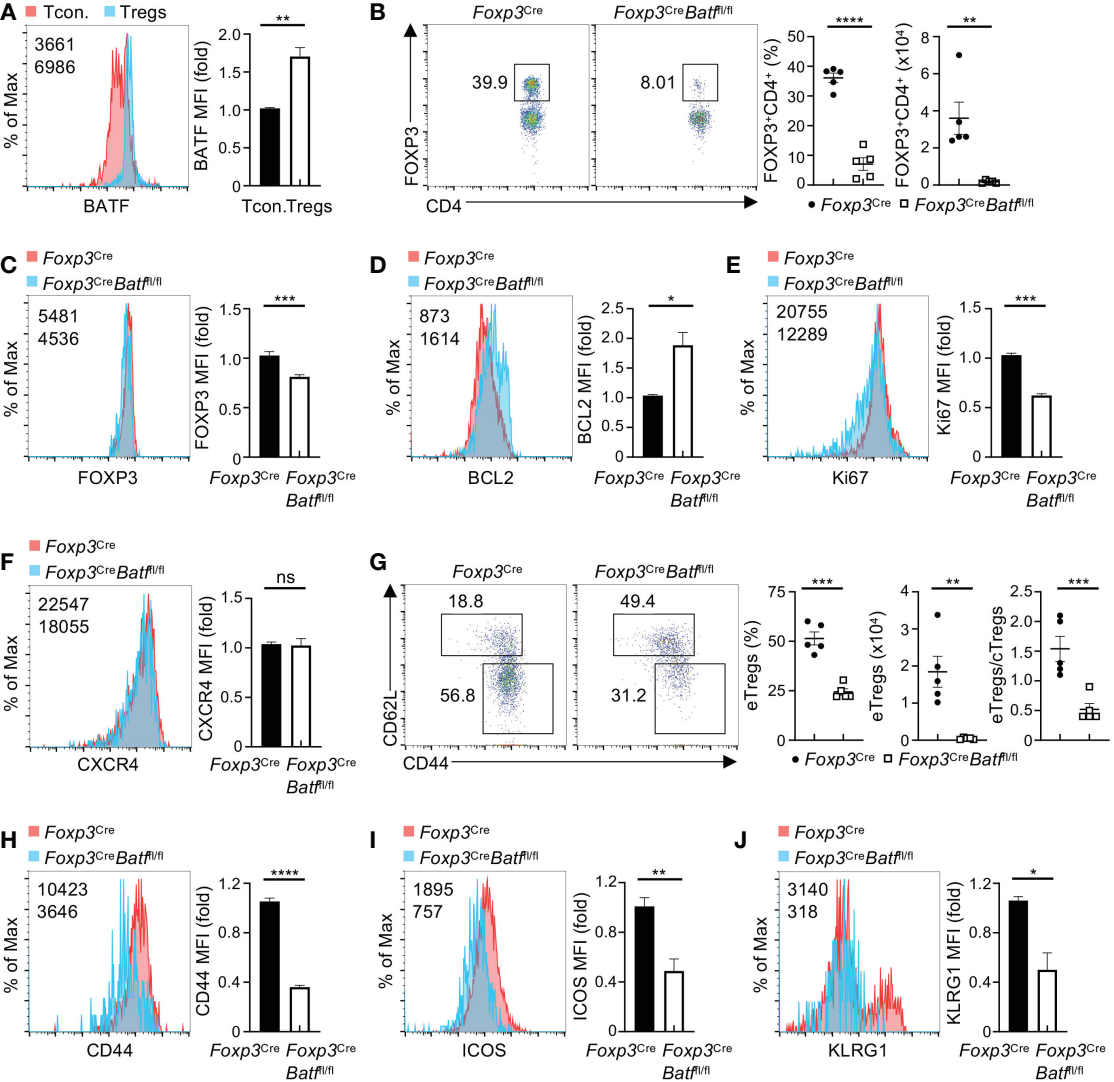
BATF deficiency compromises BM Treg homeostasis. **(A)** Flow cytometry analysis (left) of BATF expression in FOXP3^+^ Tregs and conventional FOXP3^–^CD4^+^ T (Tcon.) cells in the BM. Right, fold changes of BATF MFI in BM Tregs and Tcon. cells. **(B)** Flow cytometry analysis of FOXP3^+^ Tregs in the BM from *Foxp3* ^Cre^ and *Foxp3* ^Cre^ *Batf* ^fl/fl^ mice. Frequencies (middle) and numbers (right) of BM Tregs (*n* = 5 per group). **(C-F)** Expression of FOXP3 **(C)**, BCL2 **(D)**, Ki67 **(E)**, and CXCR4 **(F)** in *Foxp3* ^Cre^ and BATF-deficient BM Tregs. Right, fold changes of FOXP3 **(C)**, BCL2 **(D)**, Ki67 **(E)**, and CXCR4 **(F)** MFI in BM Tregs. **(G)** Flow cytometry analysis of CD62L and CD44 expression on *Foxp3* ^Cre^ and BATF-deficient BM Tregs. Frequencies (left) and numbers (middle) of BM eTregs and ratios of eTregs to cTregs in the BM (right) (*n* = 5 per group). **(H-J)** Expression of CD44 **(H)**, ICOS **(I)**, and KLRG1 **(J)** on BM Tregs from *Foxp3* ^Cre^ and *Foxp3* ^Cre^ *Batf* ^fl/fl^ mice. Right, fold changes of CD44 **(H)**, ICOS **(I)** and KLRG1 **(J)** MFI on BM Tregs. Data are representative of at least three independent experiments **(A-J)**. Data are the mean ± s.e.m. *P* values are determined by two-tailed Student’s *t*-test **(A-J)**. *P < 0.05, **P < 0.01, ***P < 0.001, and ****P < 0.0001. ns, not significant.

Recent studies highlight the importance of BATF in orchestrating homeostasis of tissue Tregs and functional specification of Tregs (17–19, 21). We thus examined whether BATF is involved in the regulation of BM Tregs. Notably, BM Tregs showed higher expression of BATF than conventional CD4^+^ T cells in the BM (**Figure 2A**). To establish the physiological relevance of BATF in BM Tregs, we examined the impact of BATF deficiency on BM Tregs from the mice with Treg-specific ablation of BATF (designated as *Foxp3* ^Cre^ *Batf* ^fl/fl^ mice) (21). Compared to *Foxp3* ^Cre^ mice, *Foxp3* ^Cre^ *Batf* ^fl/fl^ mice had largely normal numbers of total BM cells (**Figure S2A**). Strikingly, BATF deficiency profoundly abrogated frequencies and numbers of BM Tregs (**Figure 2B**), associated with diminished expression of FOXP3 (**Figure 2C**). However, the proportions of splenic Tregs in *Foxp3* ^Cre^ and *Foxp3* ^Cre^ *Batf* ^fl/fl^ mice were comparable (**Figure S2B**), implying that BM Tregs unlike splenic Tregs rely on BATF to maintain their homeostasis. To exclude the possibility that loss of BATF impairs Treg egress from the spleen in *Foxp3* ^Cre^ *Batf* ^fl/fl^ mice, we examined the proportions of Tregs in non-lymphoid tissues including the liver and colon. We found that BATF deficiency did not significantly affect the frequencies and numbers of Tregs in the liver (**Figure S2C**) and lung (**Figure S2D**) from *Foxp3* ^Cre^ *Batf* ^fl/fl^ mice at steady state. In contrast, colon Tregs from *Foxp3* ^Cre^ *Batf* ^fl/fl^ mice were markedly reduced compared to those from *Foxp3* ^Cre^ mice (**Figure S2E**), consistent with previous notion that BATF promotes migration of Tregs into the colon (15). Moreover, BATF-deficient BM Tregs showed increased expression of BCL2 (**Figure 2D**), but reduced expression of Ki67 (**Figure 2E**), indicative of their impaired homeostatic proliferation. In contrast, BATF-deficient BM Tregs had normal expression of CXCR4 (**Figure 2F**), which is crucial for Treg migration to the BM (25). Collectively, these data indicate that BATF promotes homeostatic proliferation of BM Tregs.

We next sought to determine whether BATF deficiency impinges upon the expression of eTreg-associated markers in BM Tregs. Compared to *Foxp3* ^Cre^ mice, *Foxp3* ^Cre^ *Batf* ^fl/fl^ mice had reduced frequencies and numbers of eTregs in the BM (**Figure 2G**), concomitant with a decreased ratio of eTregs to cTregs (**Figure 2G**). Loss of BATF led to reduced expression of CD44 on BM Tregs (**Figure 2H**), without substantially affecting the expression of CD62L (**Figure S2F**). In line with this observation, BATF-deficient BM Tregs dampened expression of other eTreg-associated markers including ICOS (**Figure 2I**) and KLRG1 (**Figure 2J**) relative to *Foxp3* ^Cre^ counterparts. *Foxp3* ^Cre^ *Batf* ^fl/fl^ BM Tregs had largely normal expression of OX40 (**Figure S2G**), CD73 (**Figure S2H**), and the nutrient transporters CD98 and CD71 (**Figure S2I**). BM Tregs express the IL-33 receptor ST2, which is required for chronic graft-versus-host disease control (26). BATF-deficient BM Tregs had increased expression of ST2 relative to WT counterparts (**Figure S2J**). However, BATF-deficient BM Tregs reduced the expression of CD150 (**Figure S2K**), which promotes the localization of BM Tregs in the HSC niche (5). In addition, WT and BATF-deficient BM Tregs had comparable expression of S1P1 (**Figure S2L**), a S1P receptor crucial for Treg egress from lymphoid tissues to non-lymphoid tissues (27). Thus, BATF is required for BM Tregs to sustain homeostasis and expression of eTreg-associated markers.

### *Foxp3* ^Cre^ *Batf* ^fl/fl^ mice maintain largely normal immune homeostasis in the BM

**Figure 3:**
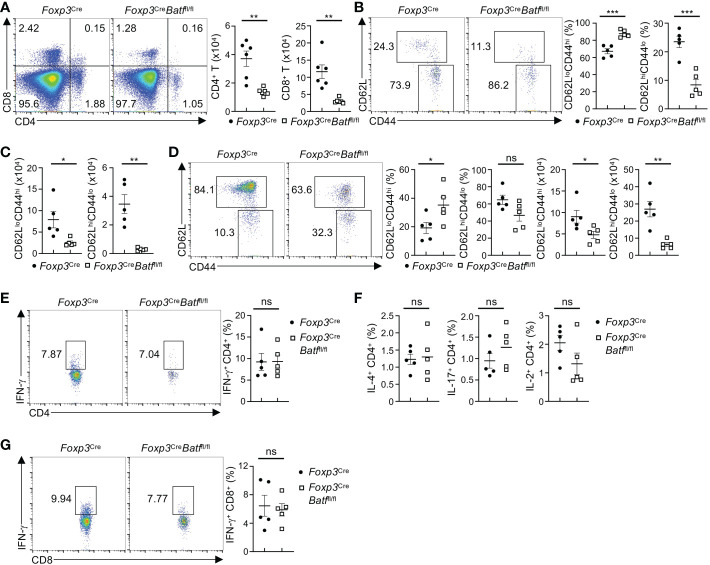
*Foxp3* ^Cre^ *Batf* ^fl/fl^ mice have reduced proportions of T cells in the BM. **(A)** Flow cytometry analysis of CD4^+^ and CD8^+^ T cells in the BM of *Foxp3* ^Cre^ and *Foxp3* ^Cre^ *Batf* ^fl/fl^ mice. Right, numbers of CD4^+^ and CD8^+^ T cells in the BM (*n* = 6 per group). **(B)** Flow cytometry analysis of CD62L and CD44 expression on conventional CD4^+^ T cells in the BM from *Foxp3* ^Cre^ and *Foxp3* ^Cre^ *Batf* ^fl/fl^ mice. Frequencies of CD62L^lo^CD44^hi^ (middle) and CD62L^hi^CD44^lo^ (right) in the BM (*n* = 5 per group). **(C)** Numbers of CD62L^lo^CD44^hi^ and CD62L^hi^CD44^lo^ CD4^+^ T cells in the BM in **(B, D)** Flow cytometric analysis of CD62L and CD44 expression on BM CD8^+^ T cells from *Foxp3* ^Cre^ and *Foxp3* ^Cre^ *Batf* ^fl/fl^ mice. Frequencies (left) and numbers (right) of CD62L^lo^CD44^hi^ and CD62L^hi^CD44^lo^ CD8^+^ T cells in the BM (*n* = 5 per group). **(E)** Flow cytometric analysis of IFN-γ production by CD4^+^ T cells in the BM from *Foxp3* ^Cre^ and *Foxp3* ^Cre^ *Batf* ^fl/fl^ mice. Right, frequencies of IFN-γ-producing CD4^+^ T cells (*n* = 5 per group). **(F)** Frequencies of BM CD4^+^ T cells producing IL-4 (left), IL-17 (middle), and IL-2 (right) from *Foxp3* ^Cre^ and *Foxp3* ^Cre^ *Batf* ^fl/fl^ mice (*n* = 5 per group). **(G)** Flow cytometry analysis of IFN-γ production by BM CD8^+^ T cells from *Foxp3* ^Cre^ and *Foxp3* ^Cre^ *Batf* ^fl/fl^ mice. Right, frequencies of IFN-γ-producing CD8^+^ T cells (*n* = 5 per group). Data are representative of at least three independent experiments **(A-G)**. Data are the mean ± s.e.m. P values are determined by two-tailed Student’s *t*-test **(A-F)**. *P < 0.05, **P < 0.01, and ***P < 0.001. ns, not significant.

We next assessed whether the reduction of BATF-deficient BM Tregs alters homeostasis of T cells in the BM. Despite BATF deficiency leading to a marked reduction of BM Tregs, *Foxp3* ^Cre^ *Batf* ^fl/fl^ mice did not show increased numbers of CD4^+^ and CD8^+^ T cells in the BM. Instead, they had diminished proportions of both CD4^+^ and CD8^+^ T cells in the BM (**Figure 3A**). Of note, *Foxp3* ^Cre^ *Batf* ^fl/fl^ mice showed reduced frequencies and numbers of naive CD4^+^ T cells (CD62L^hi^CD44^lo^) in the BM (**Figures 3B, C**). In contrast, *Foxp3* ^Cre^ *Batf* ^fl/fl^ mice had increased frequencies of effector/memory CD4^+^ T cells (CD62L^lo^CD44^hi^) (**Figure 3B**), but reduced numbers (**Figure 3C**). Similarly, *Foxp3* ^Cre^ *Batf* ^fl/fl^ BM displayed increased frequencies of effector CD8^+^ T cells (**Figure 3D**), associated with slightly reduced frequencies of naive CD8^+^ T cells (**Figure 3D**). However, numbers of both CD8^+^ T cell subsets were reduced in the *Foxp3* ^Cre^ *Batf* ^fl/fl^ BM (**Figure 3D**). Thus, loss of BATF in Tregs leads to diminished populations of BM T cells.

Given the notion that BATF-deficient Tregs fail to maintain peripheral immune tolerance (21), we examined whether cytokine production is increased in BM T cells from *Foxp3* ^Cre^ *Batf* ^fl/fl^ mice. Unexpectedly, *Foxp3* ^Cre^ *Batf* ^fl/fl^ mice exhibited largely normal proportions of BM CD4^+^ T cells producing IFN-γ (**Figure 3E**) relative to *Foxp3* ^Cre^ mice. In line with this observation, the production of IL-4, IL-17, and IL-2 by CD4^+^ T cells in the BM from *Foxp3* ^Cre^ and *Foxp3* ^Cre^ *Batf* ^fl/fl^ mice was comparable (**Figure 3F**). Moreover, *Foxp3* ^Cre^ *Batf* ^fl/fl^ BM had normal frequencies of IFN-γ-producing CD8^+^ T cells relative to *Foxp3* ^Cre^ BM (**Figure 3G**). Collectively, these results indicate that loss of BATF in Tregs does not cause uncontrolled T cell responses in the BM.

### BATF deficiency in Tregs compromises the fitness of HSCs and lineage progenitors

**Figure 4:**
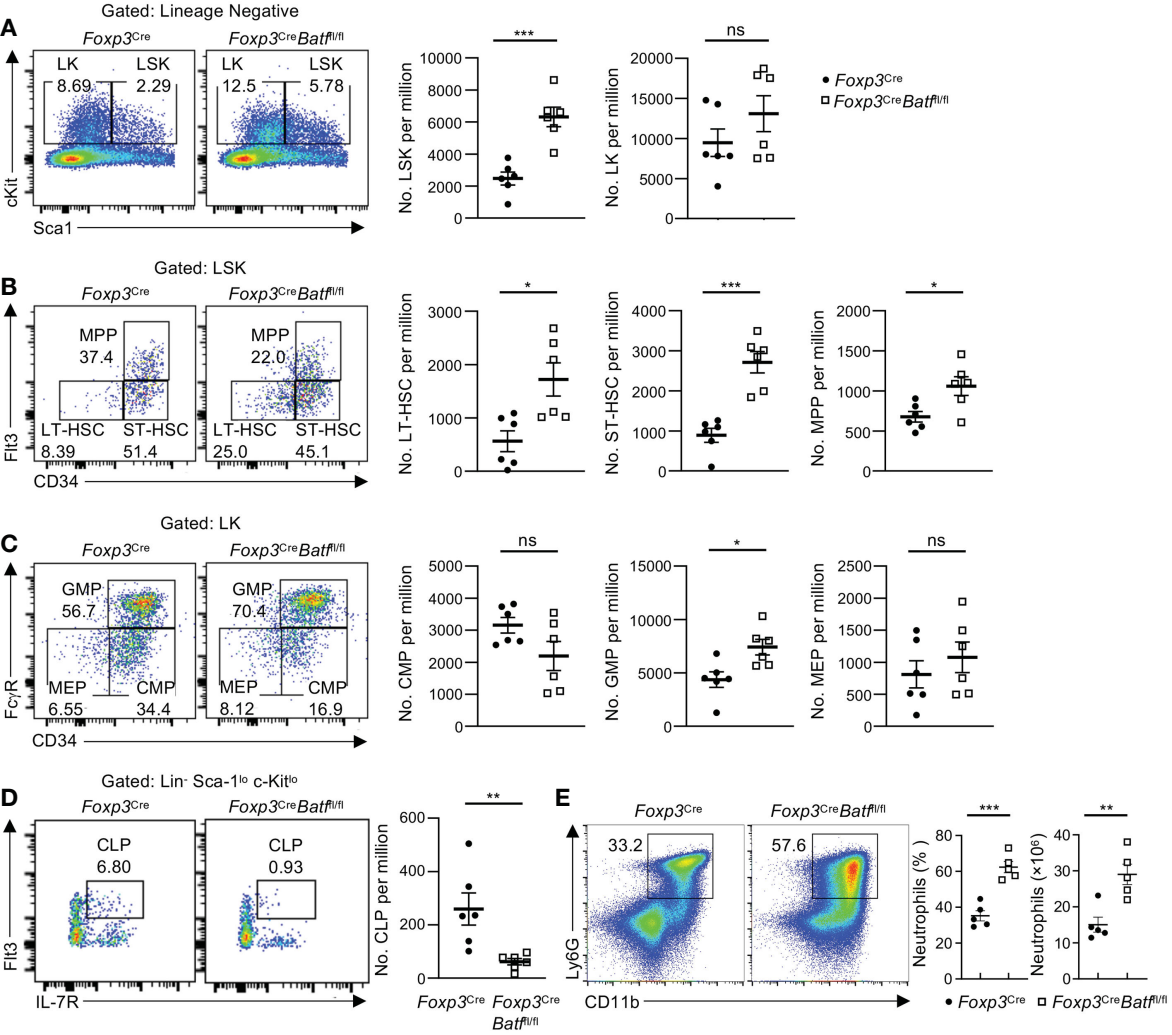
BATF deficiency in Tregs influences HSC and HPC numbers in the BM. **(A)** Flow cytometry analysis of the LSK and LK cell population in femoral BM of *Foxp3* ^Cre^ and *Foxp3* ^Cre^ *Batf* ^fl/fl^ mice. Numbers of LSK (middle) and LK cells (right) per million (*n* = 6 per group). **(B)** Flow cytometry analysis of the LT-HSC, ST-HSC and MPP cell population in femoral BM of *Foxp3* ^Cre^ and *Foxp3* ^Cre^ *Batf* ^fl/fl^ mice. Numbers of LT-HSCs (left), ST-HSCs (middle), and MPPs (right) per million (*n* = 6 per group). **(C)** Flow cytometry analysis of the CMP, GMP and MEP cell population in femoral BM of *Foxp3* ^Cre^ and *Foxp3* ^Cre^ *Batf* ^fl/fl^ mice. Numbers of CMPs (left), GMPs (middle) and MEPs (right) per million (*n* = 6 per group). **(D)** Flow cytometry analysis of the CLP population in femoral BM of *Foxp3* ^Cre^ and *Foxp3* ^Cre^ *Batf* ^fl/fl^ mice. Right, numbers of CLPs per million (*n* = 6 per group). **(E)** Flow cytometry analysis of neutrophils (CD11b^+^Ly6G^+^) in the BM from *Foxp3* ^Cre^ and *Foxp3* ^Cre^ *Batf* ^fl/fl^ mice. Frequencies (middle) and numbers (right) of neutrophils in the BM (*n* = 5 per group). Data are representative of at least three independent experiments **(A-E)**. Data are the mean ± s.e.m. *P* values are determined by two-tailed Student’s *t*-test **(A-E)**. *P < 0.05, **P < 0.01, and ***P < 0.001. ns, not significant.

It has been recently shown that BM Tregs play a crucial role in maintaining HSC homeostasis and function (3, 7, 9). As expression of YFP-Cre was undetectable in HSCs (**Figure S3A**), we next sought to determine whether the intrinsic deletion of BATF in Tregs influences HSC and hematopoietic progenitor cell (HPC) homeostasis in the BM. Loss of BATF in Tregs was associated with a significant increase in LSK (HSC-enriched) cell populations relative to *Foxp3* ^Cre^ mice (**Figure 4A**). This noticeable increase in LSK cell number within *Foxp3* ^Cre^ *Batf* ^fl/fl^ BM was associated with increased numbers of long-term HSCs (LT-HSCs), short-term HSCs (ST-HSCs), and multipotent progenitors (MPPs) compared to those in *Foxp3* ^Cre^ BM (**Figure 4B**). Despite a significant increase in the number of HSCs in *Foxp3* ^Cre^ *Batf* ^fl/fl^ BM, we did not observe any significant changes in the percentage of LT-HSCs in G0, G1, or G2/S/M phase of the cell cycle (**Figure S3B**), suggesting that there is no change in the quiescent/proliferative status of these cells. In addition, *Foxp3* ^Cre^ *Batf* ^fl/fl^ mice had a significant increase of the granulocyte-macrophage progenitor (GMP) population in the BM (**Figure 4C**), concomitant with a subsequent drop in common lymphoid progenitor (CLP) numbers (**Figure 4D**). In line with increased proportions of GMP populations, *Foxp3* ^Cre^ *Batf* ^fl/fl^ BM displayed increased frequencies and numbers of neutrophils relative to *Foxp3* ^Cre^ BM (**Figure 4E**). Taken together, these observations imply that BATF deficiency in Tregs skews the fate of HSCs towards the myeloid lineage in the BM under homeostatic conditions.

### BATF-deficient Tregs differentially influence BM and spleen HPCs

**Figure 5:**
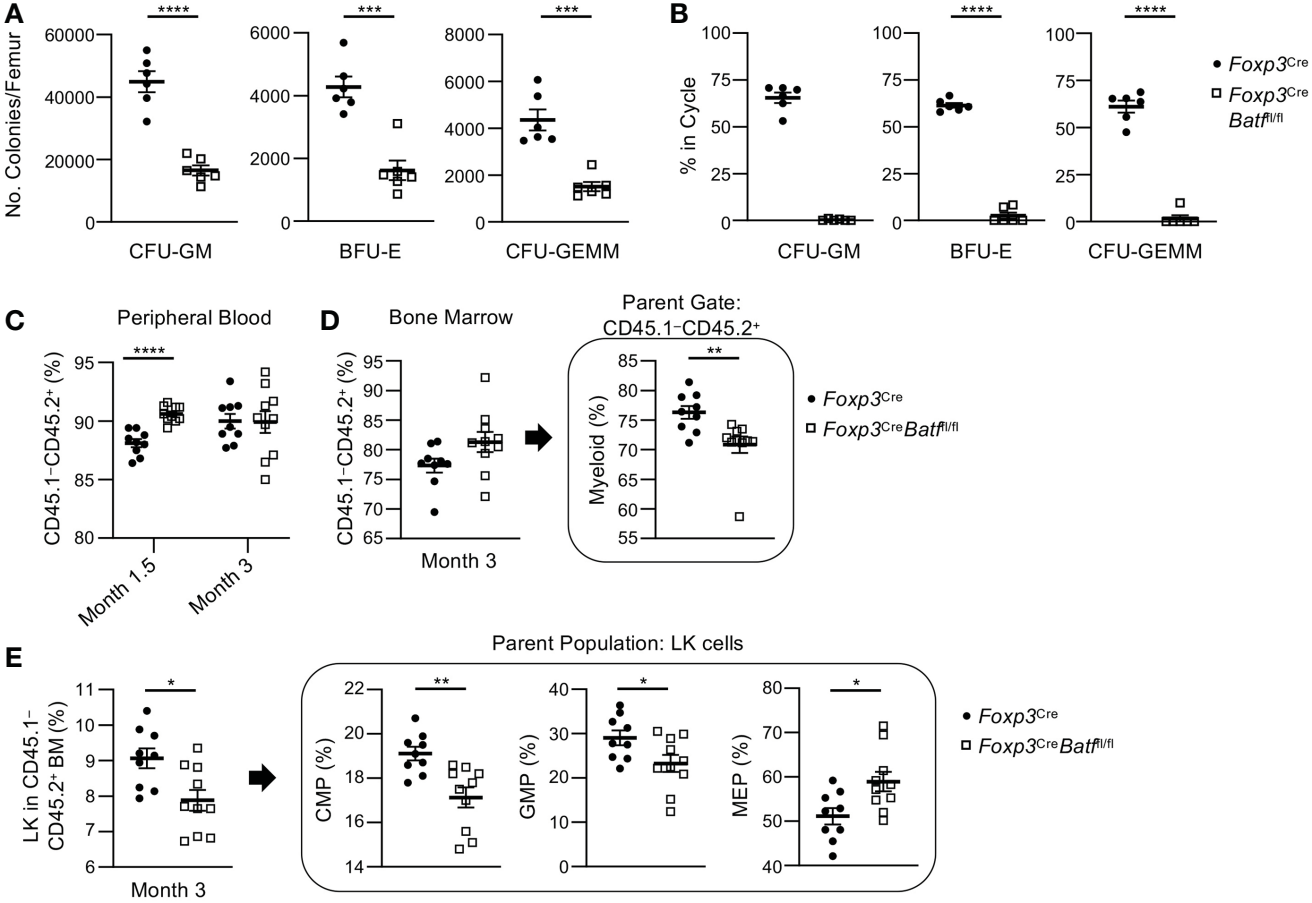
*Foxp3* ^Cre^ *Batf* ^fl/fl^ BM demonstrate a decrease in functional myeloid progenitor cells *ex vivo* and altered engraftment potential *in vivo* compared to *Foxp3^Cre^* BM. **(A)** Numbers of functional CFU-GM, BFU-E, and CFU-GEMM per femur in *Foxp3* ^Cre^ and *Foxp3* ^Cre^ *Batf* ^fl/fl^ mice (*n* = 6 per group). **(B)** Percent of CFU-GM, BFU-E, and CFU-GEMM in cycle in the BM of *Foxp3* ^Cre^ and *Foxp3* ^Cre^ *Batf* ^fl/fl^ mice (*n* = 6 per group). **(C–E)** Bone marrow transplantations were performed utilizing lethally irradiated Boy/J recipients (CD45.1^+^ CD45.2^–^) injected with a combination of donor BM (CD45.1^–^ CD45.2^+^) from *Foxp3* ^Cre^ (*n* = 9 mice per group) or *Foxp3* ^Cre^ *Batf* ^fl/fl^ (*n* = 10 mice per group) mice and support Boy/J BM cells. **(C)** Percent engraftment of donor cells (CD45.1^-^ CD45.2^+^) was examined in peripheral blood at months 1.5 and 3. **(D)** Percent engraftment and myeloid cell recovery in the bone marrow was examined 3 months post transplantation. **(E)** CMP, GMP and MEP recovery was examined within the greater myeloid progenitor cell population (Lin^–^ Sca1^–^ cKit^+^ or LK cells) 3 months post transplantation. Data are the combination of two independent experiments **(A, B)** or one experiment **(C–E)**. Data are the mean ± s.e.m. *P* values are determined by two-tailed Student’s *t*-test **(A–E)**. *P < 0.05, **P < 0.01, ***P < 0.001 and ****P < 0.0001.

It is important to note that examining immunophenotyped progenitor cell numbers does not indicate function (28). Flow cytometry is a snapshot of what is occurring in the tissue at the time of collection. To exam functional HPC populations, we performed *ex vivo* colony assays to push the differentiation/cycling of these cell populations with the addition of growth factors/cytokines, and high specific activity tritiated thymidine kill assays to determine the number of progenitors in active cycle. Colony-forming unit (CFU) assays demonstrated decreased numbers (**Figure 5A**) and cycling status (**Figure 5B**) of CFU-granulocyte-macrophage (GM), burst-forming unit-erythroid (BFU-E), and CFU-granulocyte, erythroid, macrophage, megakaryocyte (GEMM) progenitors in the BM of *Foxp3* ^Cre^ *Batf* ^fl/fl^ mice. Thus, these data demonstrate that the function and cycling of the myeloid progenitors of *Foxp3* ^Cre^ *Batf* ^fl/fl^ mice are significantly impaired, regardless of a phenotypic increase in GMP under homeostatic conditions (fresh from ‘unstimulated’ BM).

The insufficient HPC function in the BM of *Foxp3* ^Cre^ *Batf* ^fl/fl^ mice prompted us to examine function of splenic progenitor cells that can mediate extramedullary hematopoiesis in the spleen (29, 30). In contrast to *Foxp3* ^Cre^ mice, *Foxp3* ^Cre^ *Batf* ^fl/fl^ mice had increased numbers of functional CFU-GM, BFU-E, and CFU-GEMM progenitors in spleen (**Figure S4A**). Consistent with this notion, those progenitors in the spleen of *Foxp3* ^Cre^ *Batf* ^fl/fl^ mice showed enhanced cell cycling relative to counterparts in *Foxp3* ^Cre^ mice (**Figure S4B**). Collectively, the colony data from both BM and spleen indicate that knocking out BATF in Tregs results in inhibition of HPC colony formation from the BM, with a compensatory increase of HPC colony formation from the spleen.

### BM containing BATF-deficient Tregs demonstrate abnormal engraftment following transplantation

To further examine the functional differences of *Foxp3* ^Cre^ and *Foxp3* ^Cre^ *Batf* ^fl/fl^ BM HSCs and HPCs, transplantations were performed utilizing lethally irradiated Boy/J recipients (CD45.1^+^ CD45.2^–^) injected with a combination of donor BM (CD45.1^–^ CD45.2^+^) from *Foxp3* ^Cre^ (*n* = 9 mice per group) or *Foxp3* ^Cre^ *Batf* ^fl/fl^ (*n* = 10 mice per group) mice and support Boy/J BM cells. Recipient mice that received *Foxp3* ^Cre^ *Batf* ^fl/fl^ BM demonstrated significantly greater engraftment in the peripheral blood (PB) 1.5 months but not 3 months following transplantation compared to *Foxp3* ^Cre^ BM recipient mice (**Figure 5C**) suggesting that *Foxp3* ^Cre^ *Batf* ^fl/fl^ BM had better short-term but not long-term engrafting capability. Engraftment of overall donor *Foxp3* ^Cre^ and *Foxp3* ^Cre^ *Batf* ^fl/fl^ cells in the BM at 3 months showed no significant differences (**Figure 5D**). There were no significant changes in donor LT-HSC, ST-HSC and MPP percentages between *Foxp3* ^Cre^ and *Foxp3* ^Cre^ *Batf* ^fl/fl^ BM 3 months following transplantation as well (**Figure S4C**). However, engraftment of *Foxp3* ^Cre^ *Batf* ^fl/fl^ BM resulted in a decrease in mature myeloid cells (**Figure 5D**) which was reflected by a decrease in common myeloid progenitor (CMP) and GMP percentages within the overall myeloid progenitor (LK) population (**Figure 5E**). Interestingly, an increase in *Foxp3* ^Cre^ *Batf* ^fl/fl^ donor megakaryocytic-erythroid progenitor (MEP) was observed. To examine if any changes were occurring in red blood cell (RBC) and platelet recovery between recipient mice that received *Foxp3* ^Cre^ and *Foxp3* ^Cre^ *Batf* ^fl/fl^ BM, CBCs of PB were performed 3 months after transplantation (**Figure S4D-L**). Significant decreases in MCH (**Figure S4H**) and MCHC (**Figure S4I**) values and a significant increase in RDW-CV percentages (**Figure S4J**) were observed in mice that received *Foxp3* ^Cre^ *Batf* ^fl/fl^ BM suggesting that these mice are becoming or are anemic. This observation with the abnormal MEP recovery following transplantation (**Figure 5E**) and the decrease in BFU-E numbers in colony assays (**Figure 5A**) suggest abnormal erythrocyte development.

### *Foxp3* ^Cre^ *Batf* ^fl/fl^ mice display impaired B cell development

**Figure 6:**
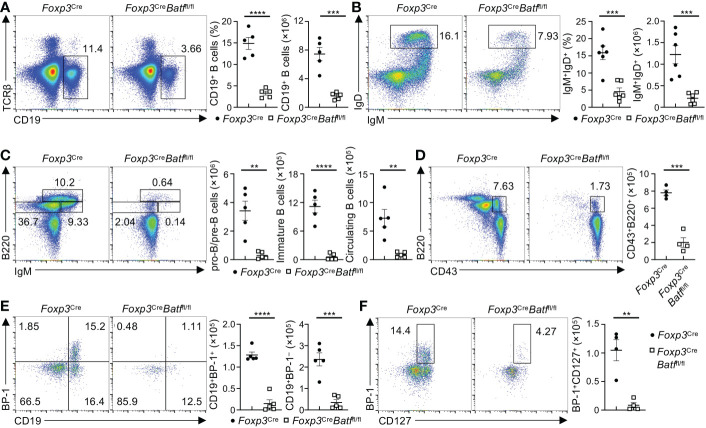
Loss of BATF in Tregs leads to the impaired development of B cells. **(A)** Flow cytometry analysis of CD19^+^ B cells in the BM from *Foxp3* ^Cre^ and *Foxp3* ^Cre^ *Batf* ^fl/fl^ mice. Frequencies (middle) and numbers (right) of CD19^+^ B cells in the BM (*n* = 5 per group). **(B)** Flow cytometric analysis of IgD^+^IgM^+^ B cells in the BM from *Foxp3* ^Cre^ and *Foxp3* ^Cre^ *Batf* ^fl/fl^ mice. Right, frequencies and numbers of IgM^+^IgD^+^ B cells (*n* = 6 per group). **(C)** Flow cytometry analysis of B220 and IgM expression in Lin^–^ BM cells from *Foxp3* ^Cre^ and *Foxp3* ^Cre^ *Batf* ^fl/fl^ mice. Right, numbers of pro-B/pre-B cells (Lin^–^B220^low^IgM^–^), immature B cells (Lin^−^B220^low^IgM^+^), and circulating mature B cells (Lin^–^B220^hi^IgM^+^) (*n* = 5 per group). **(D)** Flow cytometry analysis of B cell precursors (Lin^–^B220^+^CD43^+^IgM^–^) in the BM from *Foxp3* ^Cre^ and *Foxp3* ^Cre^ *Batf* ^fl/fl^ mice. Right, numbers of B cell precursors (*n* = 5 per group). **(E)** Flow cytometry analysis of pro-B cells (Lin^–^B220^+^CD43^+^IgM^–^CD19^+^BP-1^+/–^) in the BM from *Foxp3* ^Cre^ and *Foxp3* ^Cre^ *Batf* ^fl/fl^ mice. Right, numbers of CD19^+^BP-1^+^ and CD19^+^BP-1^–^ pro-B cells (*n* = 5 per group). **(F)** Flow cytometry analysis of CD127 and BP-1 expression on pro-B cells from *Foxp3* ^Cre^ and *Foxp3* ^Cre^ *Batf* ^fl/fl^ mice. Right, numbers of BP-1^+^CD127^+^ pro-B cells (*n* = 4 per group). Data are representative of at least three independent experiments **(A–F)**. Data are the mean ± s.e.m. *P* values are determined by two-tailed Student’s *t*-test **(A–F)**. **P < 0.01, ***P < 0.001, and ****P < 0.0001.

BM Tregs have been recently shown to support B cell development in the BM (9). To explore the potential impact of intrinsic deletion of BATF in Tregs on B cell development, we examined subpopulations of B cells in the BM (31), which did not show expression of YFP-Cre (**Figure S5A**). Of note, *Foxp3* ^Cre^ *Batf* ^fl/fl^ BM had markedly reduced frequencies and numbers of CD19^+^ B cells (**Figure 6A**) and IgD^+^IgM^+^ mature B cells (**Figure 6B**) relative to *Foxp3* ^Cre^ BM. Given the reduction of CLPs in *Foxp3* ^Cre^ *Batf* ^fl/fl^ BM (**Figure 4D**), we hypothesized that BATF deficiency in Tregs disrupts the development of B cells in the BM. To test this idea, we examined B cell subsets at the different developmental stages. IgM and B220 are well-established markers used to define B cell subsets at different development stages (32), including pro-B/pre-B cells (IgM^–^B220^low^), immature B cells (IgM^+^BM220^low^), and circulating mature B cells (IgM^+^B220^hi^). Strikingly, the frequency and number of pro-B/pre-B cells were all severely diminished in *Foxp3* ^Cre^ *Batf* ^fl/fl^ mice compared to *Foxp3* ^Cre^ counterparts (**Figure 6C**), with a concomitant reduction of immature B cells and circulating mature B cells (**Figure 6C**). We next assessed whether BATF-deficient Tregs disrupt the population of B220^+^CD43^+^IgM^–^ B cell precursors at early B cell development. Notably, *Foxp3* ^Cre^ *Batf* ^fl/fl^ BM had a significant reduction of B220^+^CD43^+^IgM^–^ B cell precursors (**Figure 6D**). B cell precursors can be further divided into different subsets based on the expression of CD19 and BP-1 (32), including pre-pro-B cells (CD19^–^BP-1^–^) and pro-B cells (CD19^+^ BP-1^+/–^). We found that the proportions of pro-B cells including CD19^+^BP-1^+^ and CD19^+^BP-1^–^ subsets were dampened in *Foxp3* ^Cre^ *Batf* ^fl/fl^ BM (**Figure 6E**), associated with increased proportions of pre-pro-B cells (**Figure 6E**). Moreover, *Foxp3* ^Cre^ *Batf* ^fl/fl^ BM showed abrogated numbers of pro-B cells expressing CD127 (**Figure 6F**), which is important for pro-B cell expansion (32). Consistent with the impaired development of B cells in the BM, both frequencies and numbers of mature B cells in the spleen from *Foxp3* ^Cre^ *Batf* ^fl/fl^ mice were diminished compared to *Foxp3* ^Cre^ counterparts (**Figure S5B**), regardless of their comparable frequencies of IgM^+^IgD^+^ B cells (**Figure S5C**). Taken together, these data indicate that BATF ensures BM Treg function to support early B cell development in the BM.

### BATF promotes the responsiveness of BM Tregs to IL-7 in maintaining homeostasis and function

**Figure 7:**
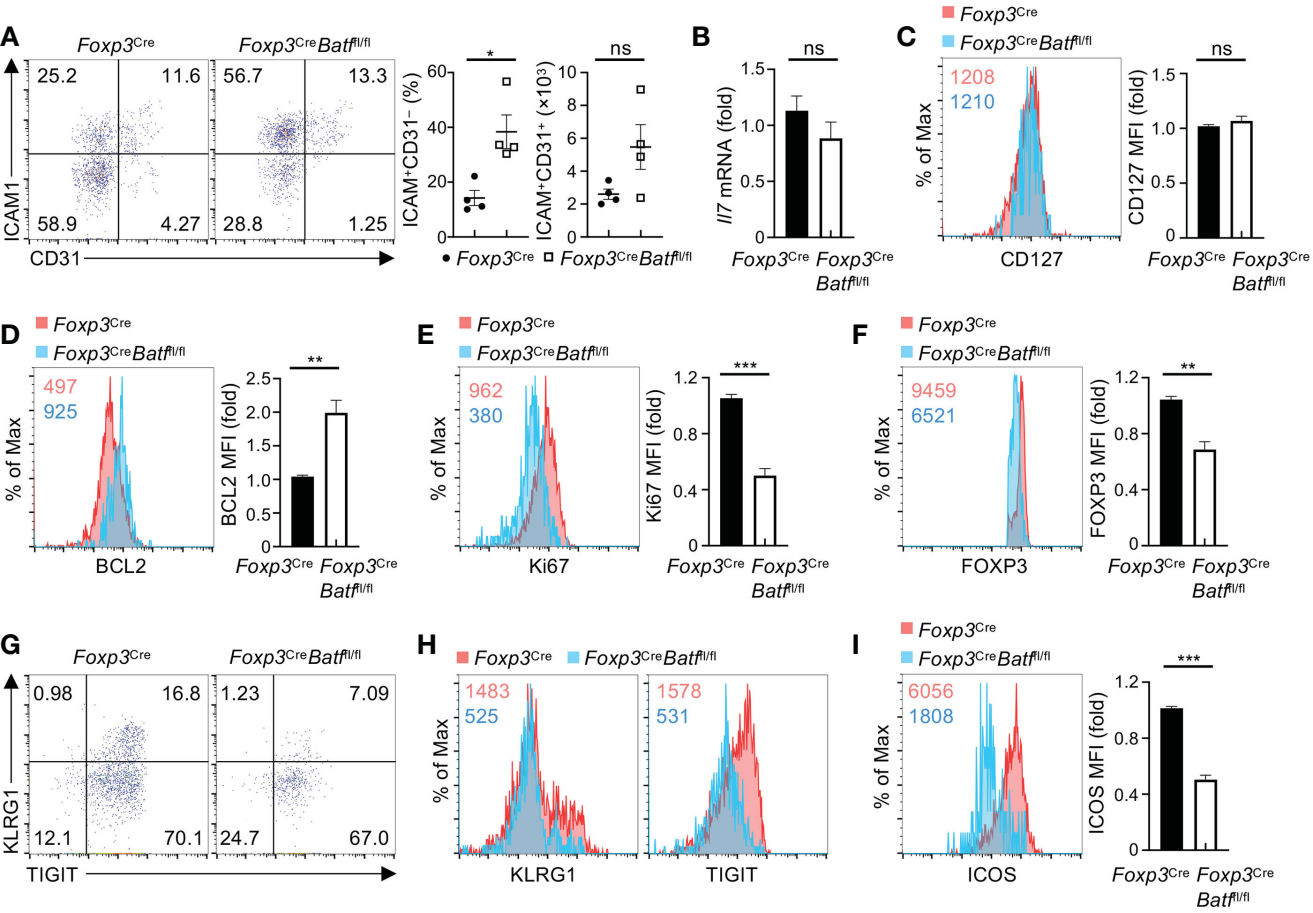
BATF promotes BM Treg homeostasis and expression of eTreg-associated markers in response to IL-7. **(A)** Flow cytometry analysis of CD31 and ICAM1 expression on BM stromal cells from *Foxp3* ^Cre^ and *Foxp3* ^Cre^ *Batf* ^fl/fl^ mice. Frequencies (middle) and numbers (right) of ICAM1^+^CD31^–^ stromal cells (*n* = 4 per group). **(B)** Relative expression of *Il7* mRNA in lineage-negative BM cells enriched from *Foxp3* ^Cre^ and *Foxp3* ^Cre^ *Batf* ^fl/fl^ mice. **(C)** Flow cytometry analysis of CD127 expression on *Foxp3* ^Cre^ and BATF-deficient BM Tregs. Right, fold changes of CD127 MFI on BM Tregs. **(D, E)** Flow cytometry analysis of BCL2 **(D)** and Ki67 **(E)** expression in *Foxp3* ^Cre^ and BATF-deficient BM Tregs stimulated with IL-7 for 48 h. Right, fold changes of BCL2 **(D)** and Ki67 **(E)** MFI in BM Tregs. **(F)** Flow cytometry analysis of FOXP3 expression of *Foxp3* ^Cre^ and BATF-deficient BM Tregs upon IL-7 stimulation. Right, fold changes of FOXP3 MFI on BM Tregs. **(G)** Flow cytometry analysis of KLRG1 and TIGIT expression on *Foxp3* ^Cre^ and BATF-deficient BM Tregs stimulated with IL-7. **(H)** Expression of KLRG1 and TIGIT MFI on IL-7-stimulated *Foxp3* ^Cre^ and BATF-deficient BM Tregs in **(G, I)** Flow cytometry analysis of ICOS expression on *Foxp3* ^Cre^ and BATF-deficient BM Tregs upon IL-7 stimulation. Right, fold changes of ICOS MFI on BM Tregs. Data are representative of at least three independent experiments **(A-I)**. Data are the mean ± s.e.m. *P* values are determined by two-tailed Student’s *t*-test **(A-F, I)**. *P < 0.05, **P < 0.01, and ***P < 0.001. ns, not significant.

Having established the importance of Treg BATF in maintaining homeostatic regulation of hematopoiesis and B cell development, we next explored the molecular mechanism underlying BATF-dependent regulation of BM Tregs. IL-7 plays a crucial role in orchestrating unique niches underlying B cell development and HSC maintenance. Acute depletion of BM Tregs has been shown to compromise a subpopulation of ICAM1^+^CD31^−^ perivascular stromal cells that produce IL-7 to fuel B cell lymphogenesis (9). We thus assessed whether BATF-deficient BM Tregs influence the population of ICAM1^+^CD31^−^ stromal cells and consequently their IL-7 production in the BM. Notably, BATF deficiency led to increased frequencies of ICAM1^+^CD31^−^ stromal cells (**Figure 7A**), without significantly affecting numbers of ICAM1^+^CD31^−^ stromal cells (**Figure 7A**). In line with this notion, expression of *Il7* mRNA was comparable in lineage-negative BM cells enriched from *Foxp3* ^Cre^ and *Foxp3* ^Cre^ *Batf* ^fl/fl^ mice (**Figure 7B**). Thus, these data imply that BATF deficiency may influence the responsiveness of BM Tregs to microenvironmental IL-7.

We next sought to assess whether BATF promotes the IL-7 responsiveness of BM Tregs in maintaining homeostasis and expression of eTreg-associated markers. To test this idea, we first examined whether BATF deficiency affects expression of IL-7 receptor CD127 on BM Tregs. Compared to *Foxp3* ^Cre^ BM Tregs, BATF-deficient BM Tregs showed comparable expression of CD127 (**Figure 7C**). Second, we assessed the impact of BATF deficiency on p-STAT5 in BM Tregs upon IL-7 stimulation. Our results showed that *Foxp3* ^Cre^ and BATF-deficient BM Tregs had comparable levels of p-STAT5 (**Figure S6A**), indicating that BATF is dispensable to induce STAT5 phosphorylation in IL-7-treated BM Tregs. Third, we examined whether BATF deficiency promotes IL-7-mediated maintenance of BM Tregs and expression of eTreg-associated markers. To this end, we cultured *Foxp3* ^Cre^ and BATF-deficient BM Tregs with IL-7 for 2 days, followed by examining BM Treg maintenance and their expression of BCL2, Ki67, and eTreg-associated markers. Of note, BATF-deficient BM Tregs showed impaired maintenance relative to BATF-sufficient BM Tregs upon IL-7 stimulation (**Figure S6B**), associated with reduced cell size (**Figure S6C**). Loss of BATF led to increased expression of BCL2 (**Figure 7D**), while reducing the expression of Ki67 (**Figure 7E**). Compared to *Foxp3* ^Cre^ BM Tregs, BATF-deficient BM Tregs showed a significant reduction of FOXP3 expression upon IL-7 stimulation (**Figure 7F**). Moreover, BATF deficiency compromised proportions of KLRG1^+^TIGIT^+^ eTregs (**Figure 7G**), associated with diminished expression of KLRG1 and TIGIT (**Figure 7H**). Consistent with these findings, expression of other eTreg-associated markers including ICOS (**Figure 7I**) and CD44 (**Figure S6D**) was abrogated on BATF-deficient BM Tregs in response to IL-7 stimulation. In contrast, BATF-deficient BM Tregs retained normal expression of CXCR4 relative to *Foxp3* ^Cre^ counterparts (**Figure S6E**). Collectively, BATF promotes the responsiveness of BM Tregs to IL-7 in sustaining BM Treg homeostasis and expression of eTreg-associated makers.

## Discussion

Aside from the importance of Tregs in maintaining immune tolerance, Tregs have been determined as crucial regulators that promote homeostasis and function of stem cells in various tissues (4). In the BM, Tregs have been shown to colocalize with HSCs in support of their persistence and function (3). Acute depletion of BM-resident Tregs disrupts homeostasis of HSCs (7) and impairs hematopoiesis of transplanted allo-HSCs (5, 26). However, the molecular mechanisms underlying BM Treg homeostasis remain unclear. In the present study, we demonstrate a previously unappreciated role for BATF in sustaining homeostasis and functionality of BM Tregs to preserve homeostatic regulation of hematopoiesis and B cell development.

Tregs integrate diverse microenvironmental cues and cellular transcriptional networks to orchestrate their homeostasis and suppressive programs in adaptation to various tissues (33). In addition to diverse local antigens, cytokines available in the tissue microenvironment play a crucial role in maintaining homeostasis and functionality of Tregs in different tissues (23). In the BM, IL-7 supports maintenance and function of HSCs (34) and promotes B cell development (35). We found that BM Tregs display higher levels of IL-7 receptor CD127 expression than splenic Tregs, associated with increased expression of FOXP3 and eTreg-associated markers such as ICOS and KLRG1. BM Tregs enhance the expression of those markers in response to IL-7 stimulation. Several lines of evidence in our studies highlight a crucial role for BATF in sustaining BM Treg homeostasis: first, specific deletion of BATF in Tregs profoundly compromises proportions of BM Tregs without substantially affecting splenic Tregs; second, loss of BATF impairs homeostatic proliferation of BM Tregs at steady state and upon IL-7 stimulation; lastly, BATF deficiency leads to reduced expression of eTreg-associated markers, such as ICOS, KLRG1, and TIGIT, on BM Tregs. In contrast, BATF-deficient BM Tregs retain normal induction of phosphorylated STAT5 upon IL-7 stimulation. Given that cooperation of BATF and STAT5 is required for Th9 cell differentiation (36), we cannot exclude the possibility that loss of BATF impairs the function of STAT5 in BM Tregs. On the basis of these observations, we conclude that BATF links microenvironmental IL-7 signal to maintaining BM Treg homeostasis.

The process of hematopoiesis, which is responsible for the production of all blood cells, is regulated by distinct BM niches that supply the requisite extrinsic factors to maintain HSC/HPC homeostasis and differentiation capacity. Recent studies highlight that BM Tregs play a pivotal role in orchestrating unique stem cell niches to enforce quiescence and function of HSCs, while the Treg intrinsic mechanisms remain largely unknown. Our studies revealed that BATF deficiency in Tregs leads to increased frequencies and numbers of HSCs, MPPs, and GMPs through an overall increase in the lineage marker negative cKit positive (an HSC and myeloid progenitor-enriched) population, associated with a reduction of CLPs, indicating that BATF-mediated support of BM Treg homeostasis and function is crucial for maintaining HSC/HPC homeostasis. However, an increase in phenotypically defined HSC/HPC numbers does not always correlate with an increase in the number of functional HSCs/HPCs (28, 37, 38). *Ex vivo* analysis demonstrated that the phenotypic increase in the indicated HSC/HPC populations in *Foxp3* ^Cre^ *Batf* ^fl/fl^ BM was not supported by a functional increase in myeloid progenitors (as determined by colony assays) as *Foxp3* ^Cre^ *Batf* ^fl/fl^ BM had markedly compromised myeloid progenitor colonization capacity. Further functional analysis of *Foxp3* ^Cre^ *Batf* ^fl/fl^ BM demonstrated that the increase in LSK cell populations seen under homeostatic conditions resulted in an increase in engraftment at 1.5 but not 3 months following transplantation, suggesting that BATF deficiency in Tregs most likely is not changing the true long-term function/self-renewal capacity of the HSC but rather the short-term engrafting capability. In addition, low myeloid progenitor and mature myeloid cell numbers in the BM with an anemic-like phenotype in the PB of *Foxp3* ^Cre^ *Batf* ^fl/fl^ BM recipient mice three months post-transplant supported our *ex vivo* observation that when ‘pushed’ *Foxp3* ^Cre^ *Batf* ^fl/fl^ BM demonstrate abnormal myelopoiesis.

BATF deficiency in Tregs is also associated with an increase in extramedullary hematopoiesis in the spleen suggesting that the spleen is compensating for the inability of the BM to maintain homeostatic levels of myelopoiesis. With a complete functional deficiency of Tregs, Scurfy mice spontaneously develop severe autoimmune and autoinflammatory responses in a variety of tissues, including liver, lung, and colon (39). Persistent inflammation in the periphery can promote extramedullary hematopoiesis in the spleen (40). Scurfy mice display excessive extramedullary myelopoiesis in the spleen, while retaining normal myelopoiesis in the BM (41). In line with the notion, *Foxp3* ^Cre^ *Batf* ^fl/fl^ mice also showed elevated extramedullary myelopoiesis in the spleen, but impaired myelopoiesis in the BM. We reasoned that the impaired myelopoiesis of *Foxp3* ^Cre^ *Batf* ^fl/fl^ BM resulted from the reduced numbers and function of BATF-deficient BM Tregs required for maintaining the HSC niches thus altering the fate decisions of these cells. Moreover, compared with scurfy mice, *Foxp3* ^Cre^ *Batf* ^fl/fl^ mice have largely normal numbers of Tregs in the spleen (21), liver, and lung, associated with modest autoimmune and autoinflammatory responses at steady state (21). However, profound autoimmunity and inflammation can influence homeostasis and function of HSCs and B cell lymphopoiesis (42, 43). Thus, we could not exclude the contribution of elevated cytokines in the periphery of *Foxp3* ^Cre^ *Batf* ^fl/fl^ mice to the compromised myelopoiesis and B cell development in the BM.

The crosstalk between BM Tregs and ICAM1^+^CD31^−^ perivascular stromal cells is crucial for B cell lymphopoiesis (9). Acute depletion of Tregs by diphtheria toxin administration compromise the population of ICAM1^+^CD31^−^ perivascular stromal cells and consequently IL-7 production, leading to the impairment of B cell lymphopoiesis. Despite that BATF deficiency leads to a marked reduction of BM Tregs, *Foxp3* ^Cre^ *Batf* ^fl/fl^ BM retains normal numbers of ICAM1^+^CD31^−^ stromal cells and IL-7 production. This discrepancy might be due to the possibility that diphtheria toxin can enable BM Tregs to undergo necroptotic cell death (44), which potentially affect the function of surrounding cells (45). *Foxp3* ^Cre^ *Batf* ^fl/fl^ BM displays diminished populations of mature B cells as well as pro-B cells, indicating that BATF deficiency in Tregs disturbs early B cell development. It is important to note that BATF deficiency leads to reduced expression of CD150 on BM Tregs, which may compromise Treg-mediated maintenance of HSC niche (34) and consequently disturb the early B cell development. The detailed mechanisms by which BATF-deficient BM Tregs impair B cell development remains to be further investigated. In addition, B cell lymphopoiesis can occur in the spleen of newborn mice and compensatory B cell production in the spleen does not occur in adult mice (46). Thus, the reduction of mature B cells in *Foxp3* ^Cre^ *Batf* ^fl/fl^ mice is resulted from the defective B cell development in the BM. Given that *Foxp3* ^Cre^ *Batf* ^fl/fl^ mice have normal numbers of splenic T cells (21), the decreased populations of BM T cells in *Foxp3* ^Cre^ *Batf* ^fl/fl^ BM are unlikely due to a developmental defect of T cells in the thymus. Collectively, BATF confer homeostasis and functionality of BM Tregs on the early development stage of B cells.

In summary, we demonstrate a previously unappreciated role for BATF that enables BM Tregs to support hematopoiesis and B cell development. BATF promotes homeostasis of BM Tregs and their expression of eTreg-associated markers in response to IL-7. BATF deficiency compromises the functionality of BM Tregs in preserving homeostasis and functional fitness of HSC/HPC populations and B cell development. Taken together, our studies provide a molecular mechanism underlying BM Treg homeostasis and functionality in the homeostatic regulation of hematopoiesis and B cell development.

## Material and methods

### Mice

C57BL/6 and Boy/J mice were purchased from the Jackson Laboratory or the breeding core facility at Indiana University School of Medicine. *Foxp3* ^YFP-Cre^ mice were gifts from A. Rudensky (47)*. Batf* ^fl/fl^ mice were kindly provided by Dr. Mark H. Kaplan. Age and gender-matched *Foxp3* ^Cre^ males or *Foxp3* ^Cre/Cre^ females and *Foxp3* ^Cre^ *Batf* ^fl/fl^ mice were generally used at 6-8-weeks old unless otherwise noted. All mice were kept in a specific pathogen-free facility in the Animal Resource Center at Indiana University School of Medicine, and all animal experiments were approved by the Institutional Animal Care and Use Committee.

### Flow cytometry

For analysis of mature immune cells, single cell suspensions from the BM and spleen were incubated with the conjugated antibodies in PBS containing 2% (vol/vol) FBS. Ghost Dye™ Violet 510 (Tonbo) was used to stain dead cells. The following antibodies were used for surface staining: CD4 (RM4-4), CD8α (53-6.7), TCRβ (H57-597), CD25 (PC61), CD44 (IM7), CD62L (MEL-14), MHC-II (M5/114.15.2), ICOS (C398.4A), CD127 (A7R34), CD98 (RL388), Ly6G (1A8), CD71 (RI7217), CD73 (TY/11.8), OX40 (OX-86), CXCR4 (L276F12), B220 (RA3-6B2), CD43 (S11), BP-1 (6C3), CD19 (6D5), IgM (RMM-1), IgD (11-26c.2a), KLRG1 (2F1/KLRG1), TIGIT (1G9), CD11b (M1/70), Siglec-F (1RNM44N), CD150 (TC15-12F12.2), ST2 (DIH9), and S1P1 (713412). Antibodies used for intracellular proteins include: IFN-γ (XMG1.2), IL-4 (11B11), IL-17 (TC11-18H10.1), BATF (D7C5), BCL2 (BCL/10C4), Ki67 (16A8), and FOXP3 (FJK-16s). All antibodies used in flow cytometry were purchased from BioLegend and Invitrogen if not otherwise indicated. For intracellular cytokine staining, T cells were stimulated for 4 h with PMA plus ionomycin in the presence of monensin before being stained according to the manufacturer’s instructions (Thermo Fisher Scientific). Flow cytometry data were acquired on the Attune NxT flow cytometer (Invitrogen) or LSR II Flow Cytometer (BD Biosciences) and analyzed with Flowjo (TreeStar). B cell subpopulations were defined as follows: circulating mature B cells (B220^hi^IgM^+^), immature B cells (Lin^−^B220^low^IgM^+^), pro-B/pre-B cells (Lin^−^B220^low^IgM^−^), B cell precursors (Lin^−^B220^+^CD43^+^IgM^−^), pre-pro-B cells (Lin^−^B220^+^CD43^+^IgM^−^CD19^−^BP-1^−^), and Pro-B cells (Lin^−^B220^+^CD43^+^IgM^−^CD19^+^BP-1^+/−^).

For analysis of HSC and hematopoietic progenitor cell (HPC) phenotypes in the BM, cells were collected at a concentration of ~3×10^6^ cells per tube, washed in PBS, incubated in a fluorescence-conjugated anti-mouse antibody cocktail for 20 minutes at room temperature (RT), washed again in PBS, and then fixed in 1.5% formaldehyde. The percentage of each cell population was used to calculate the absolute numbers of each cell population per femur. The following phenotyping markers were used: FITC- or Pacific Blue mouse lineage cocktail (CD3, Gr-1, CD11b, CD45R, Ter119; BioLegend; catalogs 133302 and 133310), PE-CF594–anti-Ly6A/E (also known as Sca-1; clone D7; BD Biosciences), APC-H7–anti-CD117 (also known as c-Kit; clone 2B8; BD Biosciences), APC– or PE–anti-CD135 (also known as Flt3; clone A2F10.1; BD Biosciences), PE– or BV421–anti-CD34 (clone RAM34; BD Biosciences), PerCP-Cy5.5–anti-CD16/CD32 (FcγII/IIIR or FcγR; clone 2.4G2; BD Biosciences), and BV421–anti-CD127 (also known as IL-7R; clone SB/199; BD Biosciences). HSC and HPC populations for mice were defined as follows- LSK cells: Lin^-^Sca-1^+^c-Kit^+^; LK cells: Lin^-^Sca-1^-^c-Kit^+^; long-term (LT)-HSCs: LSK Flt3^-^CD34^-^; short-term (ST)-HSCs: LSK Flt3^-^CD34^+^; multipotent progenitors (MPPs): LSK Flt3^+^CD34^+^; common myeloid progenitors (CMPs): LK FcγII/IIIR^lo^CD34^+^; granulocyte-macrophage progenitors (GMPs): LK FcγII/IIIR^hi^CD34^+^; megakaryocyte-erythrocyte progenitors (MEPs): LK FcγII/IIIR^–^CD34^–^; and common lymphoid progenitors (CLPs): Lin^–^Sca-1^lo^c-Kit^lo^Flt3^+^IL-7R^+^. For analysis of cell cycle status in the HSC/HPC populations, PE-anti-Ki-67 (clone B56; BD Bioscience) and DAPI (BD Biosciences) were utilized according to manufacturer’s instructions. For engraftment studies, FITC-anti-CD45.1 (clone A20; BD Bioscience) and APC-anti-CD45.2 (clone 104; BD Bioscience) were utilized. For all antibodies used in these studies, the validation for the relevant species and applications can be found on the indicated manufacturer’s website.

### Enrichment and culture of BM Tregs

BM cells were extracted from the femur and tibia. Lineage-negative (B220^−^CD11b^−^Gr-1^−^TER119^−^) BM cells were enriched with the lineage depletion kit (BD). BM Tregs were purified using CD25^+^ Treg cell isolation kit (Miltenyi). Enriched Tregs were cultured with recombinant IL-7 (50 ng/ml) in Click’s medium (plus β-mercaptoethanol) supplemented with 10% (v/v) FBS and 1% penicillin-streptomycin. At day 2 post IL-7 stimulation, BM Tregs were harvested to examine the expression of Treg associated markers.

### HPC colony and tritiated thymidine kill assays

For HPC colony assays, BM cells flushed from femurs of the indicated mice were plated at 5×10^4^ cells/mL in 1% methylcellulose culture medium with 0.1 mM hemin (MilliporeSigma), 30% FBS, 1 U/mL recombinant human erythropoietin (rhEPO) (Amgen), 50 ng/mL recombinant mouse stem cell factor (rmSCF) (R&D Systems; catalog 455-MC), and 5% vol/vol pokeweed mitogen mouse splenic cell conditioned medium. Colonies were scored after 6 days of incubation in 5% CO_2_ and lowered 5% O_2_ in a humidified chamber, and granulocyte-macrophage colony-forming units (CFU-GM), erythrocyte burst-forming units (BFU-E), and granulocyte, erythrocyte, macrophage, and megakaryocyte colony-forming units (CFU-GEMM) were distinguished by morphology of colonies. The total numbers of colonies per femur were calculated. For high specific activity tritiated thymidine kill assays, BM cells were treated with 50 μCi high specific activity [3H]Tdr (20 Ci/mmol; DuPont NEN) at RT for 40 minutes and then washed twice prior to plating for HPC colony assays (37, 38).

### Bone marrow transplantation and complete blood counts

Donor BM cells from *Foxp3* ^Cre^ or *Foxp3* ^Cre^ *Batf* ^fl/fl^ mice (CD45.1^−^ CD45.2^+^) were mixed with support BM from Boy/J mice (CD45.1^+^ CD45.2^−^) and injected intravenously into lethally irradiated (950 cGy) Boy/J mice. Percentage donor CD45.1^−^ CD45.2^+^ cells in PB were determined by flow cytometry at 1.5 and 3 months. At 3 months BM from recipient Boy/J mice was analyzed for percent donor cell engraftment, recovery of myeloid cells, and percentage of donor-derived LT-HSC, ST-HSC, MPP, CMP, GMP and MEP by flow cytometry. Complete blood counts (CBCs) were performed on recipient mouse blood collected *via* cardiac puncture at time of euthanasia utilizing a Heska Element HT5. For these experiments, cages (4–5 mice/cage) were randomly selected for transplantation group based on cage location in the cage rack. Animal studies were blinded.

### Quantitative RT-PCR

BM cells flushed from the femur and tibia were digested with collagenase IV (1 mg/ml) in RPMI 1640 medium containing 2% FBS for 45 mins. Lineage-negative (B220^−^CD11b^−^Gr-1^−^TER119^−^CD3ϵ^−^) BM cells were enriched with the lineage depletion kit (BD). RNA was extracted with microRNA isolation kit (Qiagen), and RNA concentration was determined using a NanoDrop™ One spectrophotometer (Thermo Fisher Scientific). cDNA was synthesized with SuperScript III reverse transcriptase (Invitrogen). An ABI 7500 Real-time PCR system was used for quantitative PCR with the following probe sets from Applied Biosystems: *Il7* (Mm01295803_m1) and *Actb* (Mm02619580_g1). Gene expression was normalized as n-fold difference to the housekeeping gene *Actb* as described (48).

### Statistical analysis

*P* values were calculated by two-tailed unpaired Student’s *t*-test using GraphPad Prism, unless otherwise noted. *P* values of less than 0.05 were considered as significant. *P < 0.05, **P < 0.01, ***P < 0.001, and ****P < 0.0001. All error bars represent the s.e.m.

## Data availability statement

The raw data supporting the conclusions of this article will be made available by the authors, without undue reservation.

## Ethics statement

The animal study was reviewed and approved by Matthew R. Allen.

## Author contributions

CT designed and performed cellular and molecular experiments and contributed to writing of the manuscript. LB and SG performed flow analysis, HPC colony, and BM transplant experiments. SC performed tritiated thymidine kill assay. CX, JY, and JL performed cellular experiments and data analysis. MK provided genetic models and conceptual insights. MC and KY designed experiments, wrote the manuscript, and supervised the work. All authors contributed to the article and approved the submitted version.
