# Supplementary material for: BATF sustains homeostasis and functionality of bone marrow Treg cells to preserve homeostatic regulation of hematopoiesis and development of B cells
Source: Front Immunol. 2023 Feb 22;14:1026368. doi: 10.3389/fimmu.2023.1026368 (PMC9992736; doi:10.3389/fimmu.2023.1026368)
Supplement: Supplementary file 1 [file DataSheet_1.pdf]

Supplementary Material

Supplementary Figures

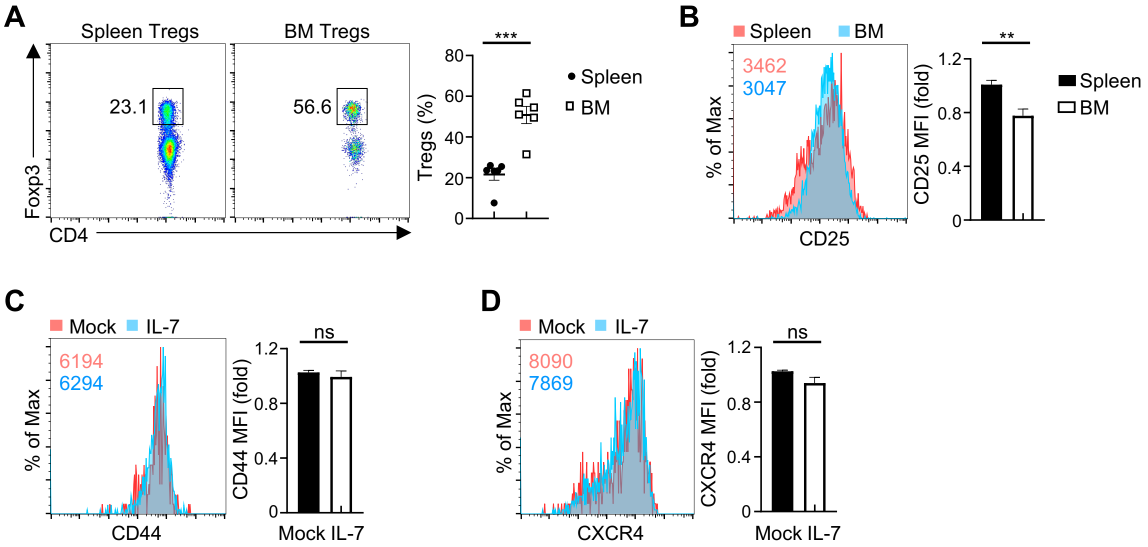

Supplementary Figure 1. The BM displays higher frequencies of Tregs than the spleen.

(**A**) Flow cytometry analysis of FOXP3$^+$ Tregs in the spleen and BM from *Foxp3*^{Cre} mice. Right, frequencies of FOXP3$^+$ Tregs in the spleen and BM (n = 6 per group). (**B**) Comparison of CD25 expression on *Foxp3*^{Cre} spleen and BM Tregs. Right, fold changes of CD25 MFI on spleen and BM Tregs. (**C, D**) Comparison of CD44 (C) and CXCR4 (D) expression on WT BM Tregs with mock or IL-7 stimulation. Right, fold changes of CD44 (C) and CXCR4 (D) MFI on BM Tregs. Data are representative of at least three independent experiments (A-D). Data are the mean ± s.e.m. *P* values are determined by two-tailed Student's *t*-test (A-D). ***P* < 0.01 and ****P* < 0.001.

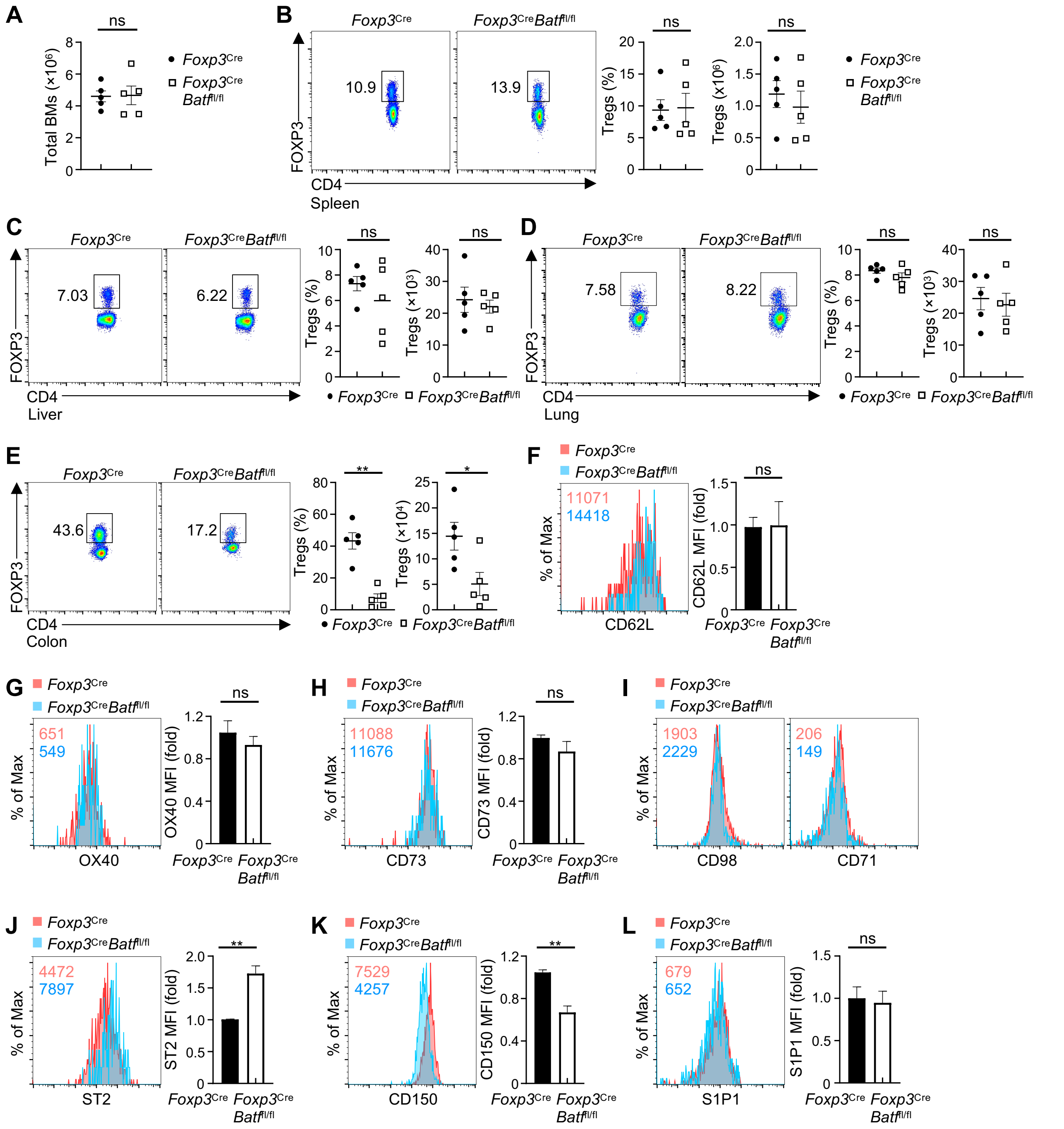

Supplementary Figure 2. The impact of BATF deficiency on tissue Tregs and expression of Treg-associated markers on BM Tregs.

(**A**) Numbers of total BMs from *Foxp3*^{Cre} and *Foxp3*^{Cre}*Batf*$^{\text{fl/fl}}$ mice (n = 5 per group). (**B**) Flow cytometry analysis of splenic FOXP3$^+$ Tregs from *Foxp3*^{Cre} and *Foxp3*^{Cre}*Batf*$^{\text{fl/fl}}$ mice (n = 5 per group). Right frequencies and numbers of splenic FOXP3$^+$ Tregs. (**C-E**) Flow cytometry analysis of FOXP3+ Tregs in the liver (C), lung (D) and colon (E) from *Foxp3*^{Cre} and *Foxp3*^{Cre}*Batf*$^{\text{fl/fl}}$ mice (n = 5 per group). Frequencies (middle) and numbers (right) of Tregs in the liver (C), lung (D), and colon (E). (**F-H**) Flow cytometry analysis of expression of CD62L (F), OX40 (G), and CD73 (H) on *Foxp3*^{Cre} and BATF-deficient BM Tregs. Right, fold changes of CD62L (F), OX40 (G), and CD73 (H) on BM Tregs

(n = 3 per group). (**I**) Flow cytometry analysis of CD98 and CD71 expression on *Foxp3*^Cre^ and BATF-deficient BM Tregs. (**J-L**) Flow cytometry analysis of ST2 (J), CD150 (K), and S1P1 (L) expression on BM Tregs from *Foxp3*^{Cre} and *Foxp3*^{Cre}*Batf*$^{fl/fl}$ mice (n = 3 per group). Right, fold changes of ST2 (J), CD150 (K), and S1P1 (L) expression on BM Tregs. Data are representative from at least three independent experiments (A-L). Data are the mean ± s.e.m. *P* values are determined by two-tailed Student's *t*-test (A-H, J-L). *$P < 0.05$ and **$P < 0.01$. ns, not significant.

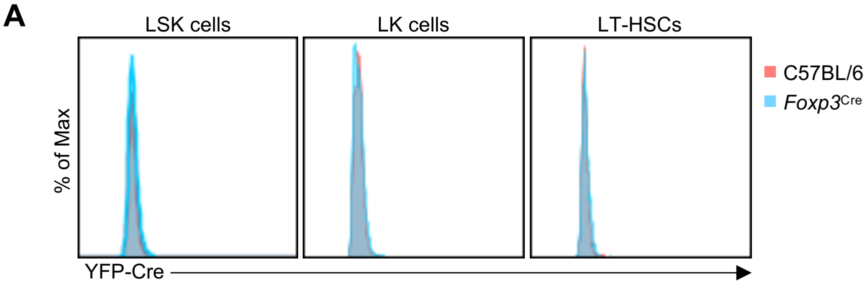

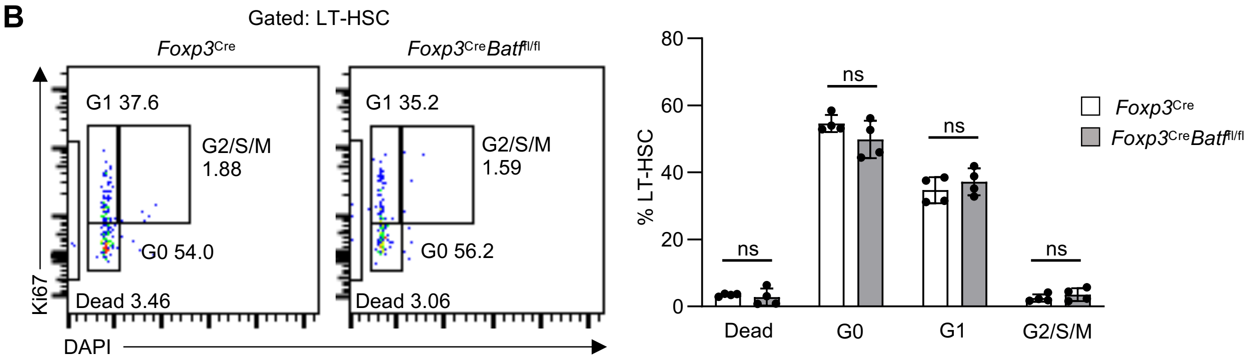

Supplementary Figure 3. There is no observed difference in HSC quiescence in the BM of *Foxp3*^{Cre}*Batf*$^{fl/fl}$ mice.

(**A**) Flow cytometry analysis of YFP-Cre expression in the populations of LSK cells, LK cells, and LT-HSCs from C57BL/6 and *Foxp3*^{Cre} mouse BM. (**B**) Flow cytometry analysis of BM LT-HSCs from *Foxp3*^{Cre} and *Foxp3*^{Cre}*Batf*$^{fl/fl}$ mice in the G0, G1, and G2/S/M phase of the cell cycle. Right, Frequencies of BM LT-HSCs in the indicated phase of the cell cycle (n = 4 per group). Data are from one (A) or three (B) independent experiments. Data are the mean ± s.e.m. *P* values are determined by two-tailed Student's *t*-test. ns, not significant.

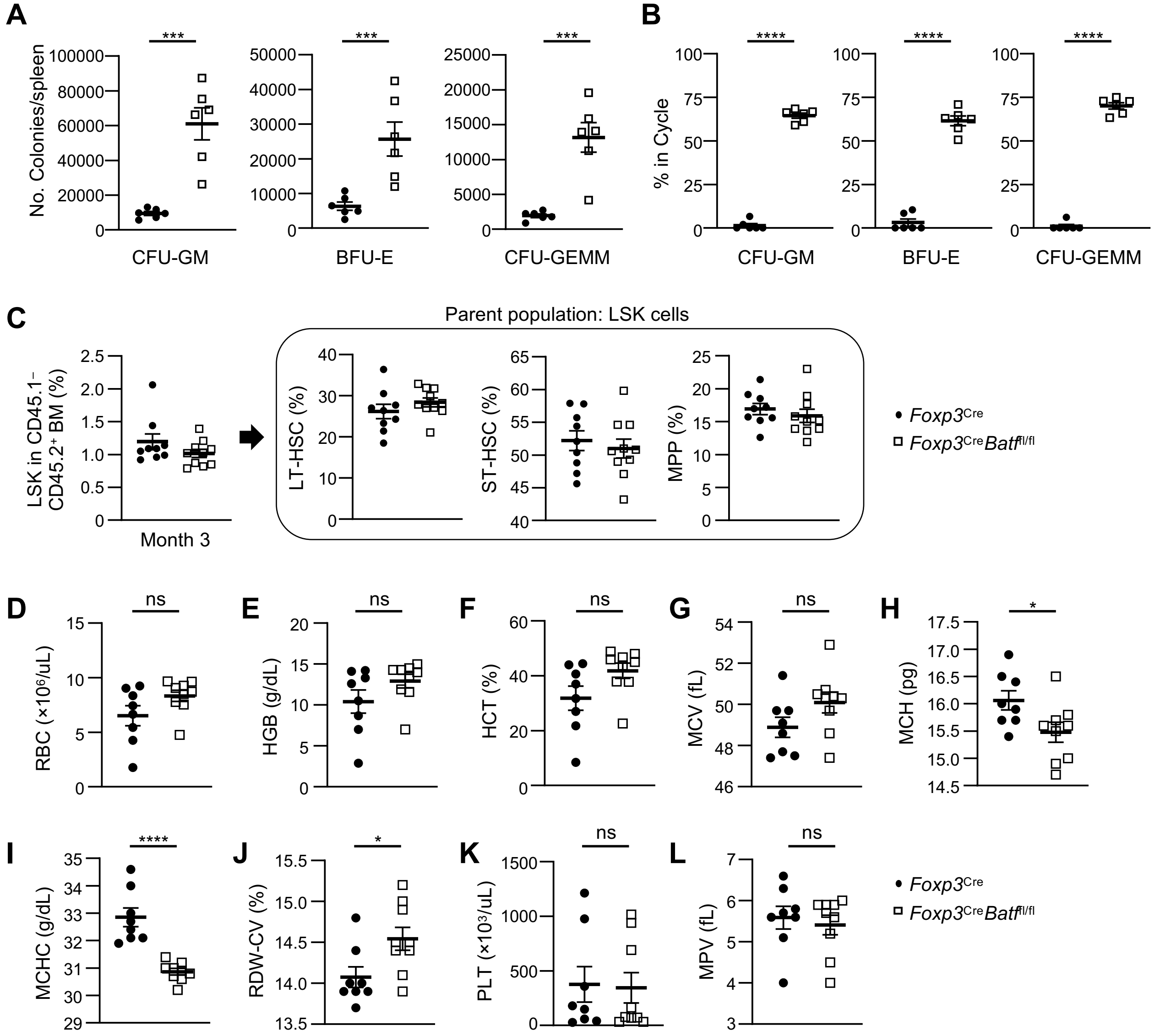

Supplementary Figure 4. *Foxp3*^{Cre}*Batf*$^{\mathrm{fl/fl}}$ mice demonstrate altered hematopoiesis both pre- and post-transplantation.

(**A**) Numbers of functional CFU-GM, BFU-E, and CFU-GEMM in the spleen of *Foxp3*^{Cre} and *Foxp3*^{Cre}*Batf*$^{\mathrm{fl/fl}}$ mice (n = 6 per group). (**B**) Percent of CFU-GM, BFU-E, and CFU-GEMM in cycle in the spleen of *Foxp3*^{Cre} and *Foxp3*^{Cre}*Batf*$^{\mathrm{fl/fl}}$ mice (n = 6 per group). (**C**) Bone marrow transplantations were performed utilizing lethally irradiated Boy/J recipients (CD45.1^{+} CD45.2^{-}) injected with a combination of donor BM (CD45.1^{-} CD45.2^{+}) from *Foxp3*^{Cre} (n = 9 mice per group) or *Foxp3*^{Cre}*Batf*$^{\mathrm{fl/fl}}$ (n = 10 mice per group) mice and support Boy/J BM cells. LT-HSC, ST-HSC and MPP recovery was examined within the Lin^{-} Sca1^{+} cKit^{+} (LSK) population 3 months post transplantation. (**D-L**) Complete blood counts were performed on the recipient mice from (C) prior to BM collection at 3 months post transplantation on a Heska Element HT5. Red blood cell (RBC) numbers (D), hemoglobin (HGB) levels (E), hematocrit (HCT; F), mean corpuscular volume (MCV; G), mean corpuscular hemoglobin

(MCH; H), mean corpuscular hemoglobin concentration (MCHC; I), red cell distribution width (RDW-CV; J), platelet count (PLT; K), and mean platelet volume (MPV; L) was measured. *Foxp3*^{Cre} (n = 8) and *Foxp3*^{Cre}*Batf*$^{fl/fl}$ (n = 9) recipient Boy/J mice. Data are the combination of two independent experiments (A, B) or one experiment (C-L). Data are the mean ± s.e.m. P values are determined by two-tailed Student's t-test (A, B, D-L). *$P < 0.05$, ***$P < 0.001$, and ****$P < 0.0001$. ns, not significant.

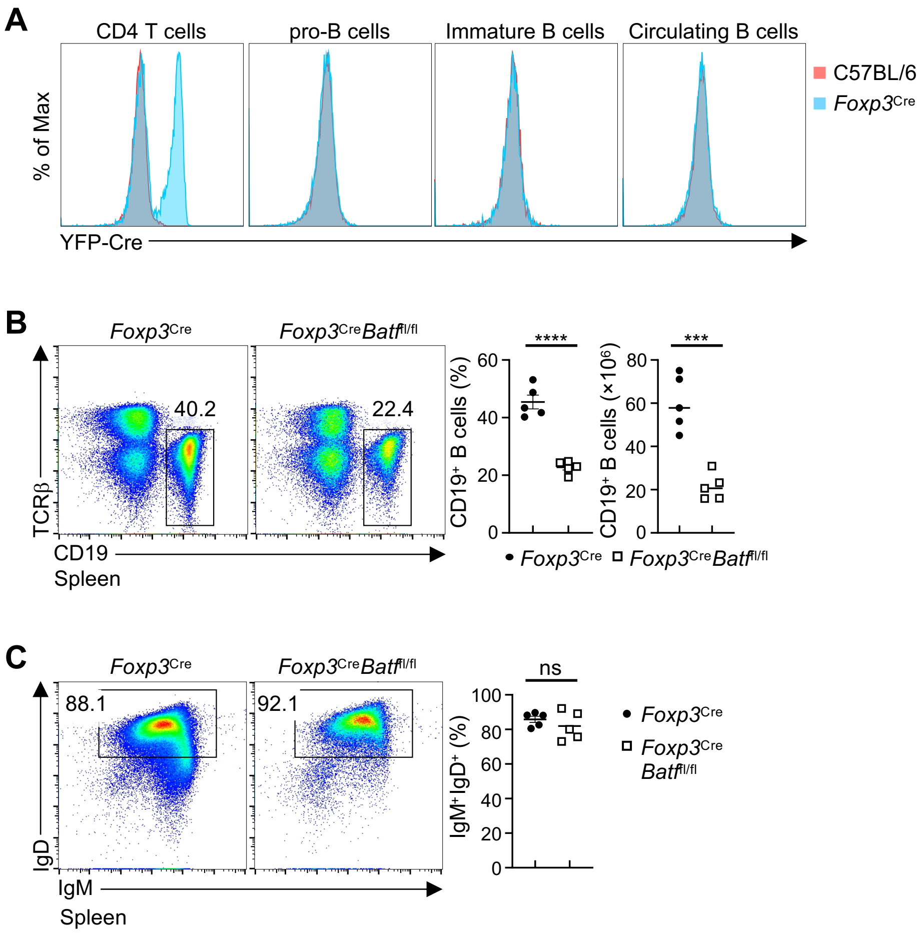

Supplementary Figure 5. The proportion of mature splenic B cells is reduced in *Foxp3*^{Cre}*Batf*$^{\mathrm{fl/fl}}$ mice.

(**A**) Flow cytometry analysis of YFP-Cre expression in the populations of CD4^{+} T cells, pro-B cells, immature B cells, and circulating B cells from C57BL/6 and *Foxp3*^{Cre} mouse BM. (**B**) Flow cytometry analysis of CD19^{+} B cells in the spleen from *Foxp3*^{Cre} and *Foxp3*^{Cre}*Batf*$^{\mathrm{fl/fl}}$ mice. Frequencies (middle) and numbers (right) of splenic CD19^{+} B cells (n = 5 per group). (**C**) Flow cytometry analysis of IgD and IgM expression on splenic CD19^{+} B cells from *Foxp3*^{Cre} and *Foxp3*^{Cre}*Batf*$^{\mathrm{fl/fl}}$ mice. Right, frequencies of splenic IgM^{+}IgD^{+} B cells (n = 5 per group). Data are representative from at least three independent experiments (A-C). Data are the mean ± s.e.m. P values are determined by two-tailed Student's t-test (B, C). ***$P < 0.001$ and ****$P < 0.0001$. ns, not significant.

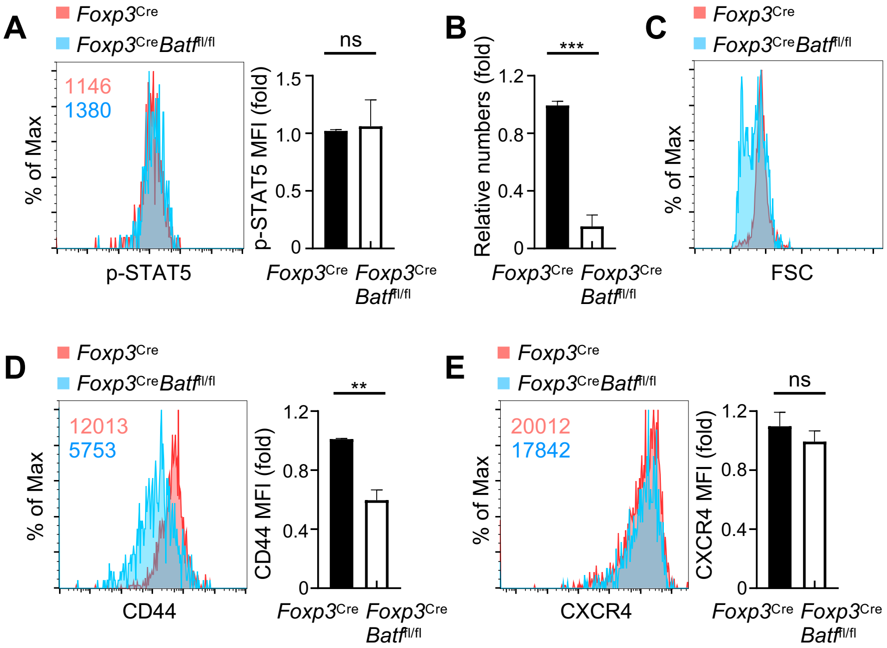

Supplementary Figure 6. The impact of BATF deficiency on BM Tregs upon IL-7 stimulation.

(**A**) Flow cytometry analysis of p-STAT5 in *Foxp3*^{Cre} and BATF-deficient BM Tregs with mock or IL-7 stimulation. Right, fold changes of p-STAT5 MFI on BM Tregs. (**B**) Relative numbers of *Foxp3*^{Cre} and BATF-deficient BM Tregs stimulated with IL-7 for 2 days. (**C**) Flow cytometry analysis of cell size of *Foxp3*^{Cre} and *Foxp3*^{Cre}*Batf*$^{\mathrm{fl/fl}}$ BM Tregs stimulated with IL-7 for 2 days. (**D**, **E**) Flow cytometry analysis of expression of CD44 (D) and CXCR4 (E) on *Foxp3*^{Cre} and BATF-deficient BM Tregs. Right, fold changes of CD44 (D) and CXCR4 (E) on BM Tregs. Data are representative from at least three independent experiments (A-E). Data are the mean ± s.e.m. *P* values are determined by two-tailed Student's *t*-test (A, B, D, E). $^{**}P < 0.01$ and $^{***}P < 0.001$. ns, not significant.
